# Study on Incentives for Glaucoma Medication Adherence (SIGMA): study protocol for a randomized controlled trial to increase glaucoma medication adherence using value pricing

**DOI:** 10.1186/s13063-016-1459-1

**Published:** 2016-07-15

**Authors:** Marcel Bilger, Tina T. Wong, Kaye L. Howard, Jia Yi Lee, Ai Nee Toh, Geraldine John, Ecosse L. Lamoureux, Eric A. Finkelstein

**Affiliations:** Health Services & Systems Research, Duke-NUS Graduate Medical School, 8 College Road, 169857 Singapore, Singapore; Singapore Eye Research Institute, Singapore National Eye Centre, 11 Third Hospital Avenue, 168751 Singapore, Singapore; Duke Global Health Institute, Duke University, 310 Trent Drive, Durham, NC 27710 USA

**Keywords:** Glaucoma, Chronic disease, Medication adherence, Financial incentive, Contingent rebates, Conditional cash transfer, Value pricing, Value-based insurance, Loss aversion

## Abstract

**Background:**

Many glaucoma patients do not adhere to their medication regimens because they fail to internalize the (health) costs of non-adherence, which may not occur until years or decades later. Behavioural economic theory suggests that adherence rates can be improved by offering patients a near-term benefit. Our proposed strategy is to offer adherence-contingent rebates on medication and check-up costs. This form of value pricing (VP) ensures that rebates are granted only to those most likely to benefit. Moreover, by leveraging loss aversion, rebates are expected to generate a stronger behavioural response than equivalent financial rewards.

**Methods/Design:**

The main objective of the Study on Incentives for Glaucoma Medication Adherence (SIGMA) is to test the VP approach relative to usual care (UC) in improving medication adherence. SIGMA is a randomized, controlled, open-label, single-centre superiority trial with two parallel arms. A total of 100 non-adherent (Morisky Medication Adherence Scale ≤6) glaucoma patients from the Singapore National Eye Centre are block-randomized (blocking factor: single versus multiple medications users) into the VP and UC arms in a 1:1 ratio. The treatment received by VP patients will be strictly identical to that received by UC patients, with the only exception being that VP patients can earn either a 50 % or 25 % rebate on their glaucoma-related healthcare costs conditional on being adherent on at least 90 % or 75 % of days as measured by a medication event monitoring system. Masking the arm allocation will be precluded by the behavioural nature of the intervention but blocking size will not be disclosed to protect concealment. The primary outcome is the mean change from baseline in percentage of adherent days at month 6. A day will be counted as adherent when the patients take all their medication(s) within the appropriate dosing windows.

**Discussion:**

This trial will provide evidence on whether adherence-contingent rebates can improve medication adherence among non-adherent glaucoma patients, and more generally whether this approach represents a promising strategy to cost-effectively improve chronic disease management.

**Trial registration:**

NCT02271269. Registered on 19 October 2014.

**Electronic supplementary material:**

The online version of this article (doi:10.1186/s13063-016-1459-1) contains supplementary material, which is available to authorized users.

## Background

### Rationale

Glaucoma refers to a group of eye conditions that lead to damage to the optic nerve, which carries information from the eye to the brain. If uncontrolled, glaucoma first causes peripheral vision loss and eventually can lead to blindness. Glaucoma affects approximately 60 million people worldwide and is one of the leading causes of irreversible blindness [1]. In Singapore, glaucoma, affects roughly 3 % of those over age 40 and the total number of cases is growing due to Singapore’s ageing population [2, 3]. The most important modifiable factors of visual field loss in glaucoma are peak intraocular pressure (IOP), average IOP and fluctuations in IOP [4–7]. The majority of patients with glaucoma or suspected glaucoma are initially managed by IOP-reducing single or multi-drug treatments consisting of topical eye drops. This could be followed by surgical treatment for those whose IOP is not adequately controlled via medications [8, 9].

Correct use of medicated eye drops reduces IOP, subsequently slowing visual field loss for nearly 90 % of patients [10–13]. Despite the effectiveness of topical medication in controlling disease progression, roughly two-thirds of patients report some level of medication non-adherence to their medication [14–16]. Interventions to enhance medication adherence often focus on one or a combination of four strategies: simplification, education, social support and behaviour modification [17]. Most simplification interventions focus on reducing the number of doses per day, perhaps through extended-release capsules, or the number of medications [17]. Simplifying dosage requirements has consistently been shown to improve adherence [17–19]. However, there is evidence of non-adherence amongst glaucoma patients taking only one topical medication [20]. Education and social support interventions aim to increase adherence through greater knowledge transfer and increased self-efficacy. Education and social support interventions have shown some evidence of effectiveness in the short term, but results are less compelling in the long term [21, 22]. Behavioural interventions cover a wide range of strategies, such as pill organizers, reminder systems, and tailored regimens [17]. These studies too have shown mixed results, with both positive and negative results relating to the effectiveness of reminders, and no agreement on which behavioural intervention works best [23–26]. In a review of randomized control trials that seek to improve medication adherence, those that were most effective were multi-faceted, and included combinations of convenience, education, reminders, and reinforcement [19]. However, even the most effective interventions reviewed did not lead to large improvements in adherence. As a result, other strategies are needed.

Behavioural economics theory suggests that an important factor of non-adherence is that patients do not perceive a clear cause-and-effect relationship between non-adherence and the increased likelihood of disease progression, which may not occur until well into the future [27, 28]. As a result, many patients do not internalize the consequences of non-adherence until it is too late. One strategy to rectify this problem is to provide a short-term reward for increased adherence. Giuffrida et al. [29] reviewed 11 randomized incentive trials conducted in the United States where patients were paid either cash, gifts or vouchers for meeting adherence targets to various treatments and health services. These rewards, which ranged in value from USD5 to nearly USD1000, showed improve adherence in 10 out of the 11 studies reviewed. However, none focused on glaucoma patients and results varied widely across studies, suggesting that more research is needed to identify an optimal strategy to cost-effectively improve medication adherence [30].

In this trial we test a novel approach to improve medication adherence among glaucoma patients. The approach consists of adherence-contingent rebates on medication and check-up costs that are granted only when adherence goals are met as verified by a medication event monitoring system. Given that prescription refills and follow-up clinic visits occur regularly, the rebates provide a tangible and near-term benefit resulting from medication adherence. This strategy, which has been suggested by Loewenstein and colleagues [31], can be seen as a novel form of value pricing (VP) in the context of value-based insurance designs [32]. With standard value-based insurance designs, the co-payment for clinically effective treatments is reduced in efforts to increase their utilization. With our intervention design, incentives are allocated to medications that have not only been shown to be clinically effective, but that are also being effectively used by the patient, which represents a better use of resources. Another important feature of our approach is that incentives are provided in the form of rebates on costs already incurred by the patient. By offering a rebate that avoids a loss, as opposed to an equally sized reward, loss aversion theory predicts that this approach is likely to have a greater behavioural response [33].

This trial will provide evidence on whether adherence-contingent rebates can improve medication adherence among non-adherent glaucoma patients. Secondary objectives are to determine whether IOP and quality of life can also be improved, and whether the intervention represents a promising strategy to cost-effectively improve glaucoma management. Finally, explanatory analysis will aim at uncovering factors that might moderate the intervention effect and explain medication adherence.

### Objectives

*Primary objective*: determine whether complementing usual care (UC) with adherence-contingent rebates according to a VP strategy is superior to UC alone in improving medication adherence between baseline and month 6.

*Secondary objective 1*: determine whether the IOP of patients in the VP arm improves more (or deteriorates less) than that of patients in the UC arm between baseline and month 6.

*Secondary objective 2*: determine whether the glaucoma-related (GQL-15) and generic health-related (EQ5D-5 L) quality of life of patients in the VP arm improves more (or deteriorates less) than that of patients in the UC arm between baseline and month 6.

*Secondary objective 3*: determine whether the incremental cost-effectiveness ratio (ICER) of VP compared to UC will be favourable relative to international benchmarks for cost-effectiveness.

### Explanatory analyses

(i) compare adherence levels and intervention effect according to different definitions (aspects) of medication adherence
(ii) determine factors that might moderate the intervention effect on medication adherence and quality of life
(iii) determine factors of medication adherence at baseline

## Methods

### Trial design

The Study on Incentives for Glaucoma Medications Adherence (SIGMA) trial is designed as a randomized, controlled, open-label, single-centre superiority trial with two parallel arms. A total of 100 non-adherent glaucoma patients will be block-randomized (blocking factor: single versus multiple medication users) into the UC and VP arms in a 1:1 ratio. Baseline assessment lasts 1 month, followed by a 5-month intervention. The primary outcome is medication adherence as recorded by an electronic medication container.

### Study setting and eligibility criteria

All patients will be recruited at the Singapore National Eye Centre (SNEC). Singapore is an island city-state that has a population of 5.5 million and which is one of the most densely populated countries in the world. Singapore is a multicultural country consisting of Chinese (76.2 %), Malay (15 %), Indian (7.4 %) and other (1.4 %) ethnicities. The most widely spoken languages in Singapore are English, Mandarin, Malay and Tamil. The vast majority of those who speak Malay and Tamil are also fluent in English.

SNEC is part of SingHealth, which is Singapore’s largest public healthcare group. Since 1990, SNEC has been providing a full range of high-quality eye care and is a referral centre for complex cases both nationally and internationally. With its research arm, the Singapore Eye Research Institute (SERI), SNEC also extensively engages in academic research. SNEC treats both high-income patients and low-income patients. All Singaporean citizens and permanent residents are entitled to government subsidies in case of referral, while low-income patients can benefit from additional subsidies through various government schemes. The study will be conducted in collaboration with Duke-NUS, which is part of the academic medical centre SNEC belongs to.

#### Inclusion criteria

- Glaucoma patient taking at least one eye drop medication
- Low medication adherence based on the 8-Item Morisky Medication Adherence Scale (MMAS) [34–36]
- Aged between 21 and 85 years
- Singaporean citizens or permanent residents
- Conversant in English or Mandarin

#### Exclusion criteria

- Stage 4 (severe) or Stage 5 (end-stage) glaucoma according to the Glaucoma Staging System (GSS) based on the Humphrey Visual Field [37].
- Patients who are not independently instilling their medications all or most of the time.

### Study arms

In this study, patients learn about their arm allocation by phone 1 month after their enrolment. This is to collect 1 month of baseline data on medication adherence. The patients then remain in their respective study arm for a duration of 5 months. In both arms, a questionnaire and clinical assessment will take place at baseline and month 6.

**Fig. 1:**
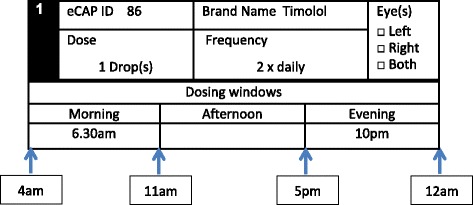
Glaucoma medication dosing schedule. Note: Window length is set at a minimum of 6 hours and a maximum of 8 hours in the morning, afternoon or evening

During the baseline visit, the research optometrist takes note of the glaucoma medication dosing schedule (see Fig. 1) in the participant instruction booklet (see Additional file 1) to ensure a uniform presentation of adherence goals across participants. Medication adherence will be monitored during the whole 6-month period by means of electronic containers eCAPs™ (Information Mediary Corporation, Ottawa, ON, Canada). An eCAP™ is a medication event monitoring system with an inbuilt electronic tag which records the time whenever it is opened and closed. Each eye drop will be stored in separate vials with separate eCAPs™. At month 3, eCAPs™ are collected by a courier service, medication adherence is calculated for months 2 and 3 and the eCAPs™ are returned to the patients along with a short adherence report for that period (see Additional files 2 and 3). The next assessment period for medication adherence runs from months 4 to 6, and adherence calculation takes place after the patients returned their eCAPs™ at the month 6 study assessment. Patients also receive a call at the end of months 2 and 5 to verify that they understand their arm allocation and adherence goals, and that patients with multiple eye drops keep their medication in the assigned eCAP™.

#### Arm 1: usual care (UC)

Patients attend routine check-ups with an ophthalmologist where they have glaucoma eye drops prescribed for them, which they then purchase at the SNEC pharmacy. The frequency of check-ups varies between patients depending on the severity of their condition. The decision is made by the ophthalmologist on how regularly patients should return for check-ups, ranging from 3 months to once a year. IOP and visual acuity are measured at every visit while visual field readings are taken annually. Non-adherent patients may also be offered glaucoma counselling if necessary. During a counselling session, a nurse delivers an educational component on effective glaucoma treatment. Counselling includes the following components: (i) understanding of glaucoma and visual field process with a focus on risk factors and symptoms, (ii) management and treatment of glaucoma, (iii) medications usage with practical advice on self-application of eye drops. In addition, the nurse counsellor determines a dosing schedule for each medication that accommodates the patient’s lifestyle and takes into account working hours. During this discussion, the nurse counsellor underlines the health risks raised by non-adherence to the medication regimen.

In order to properly identify the effect of value pricing, we brought the following deviations to usual care. First, a short adherence report will be sent to the participants as described above. Second, a non-contingent payment of SGD30 will be made to them at months 3 and 6. However, unlike VP patients, no financial incentive will be given when they are adherent to their medications.

#### Arm 2: value pricing (VP)

The treatment received by VP patients is strictly identical to that received by UC patients, with the only exception being that VP patients can earn rebates on their glaucoma-related healthcare costs contingent on being sufficiently adherent to their medications.

Adherence is measured as the percentage of days where the patients take all their medication(s) within the appropriate dosing windows for the day. According to this definition, if a patient fails to take a single dose during the corresponding window, the whole day is counted as non-adherent. This concept of adherent-day stresses the importance of complying to the medication regimen as a whole and to its schedule in efforts to limit detrimental IOP fluctuations.

Patients who achieve more than 90 % adherent days will receive a rebate amounting to 50 % of their glaucoma-related healthcare costs, and patients achieving between 75 % and 90 % of adherent days will receive a 25 % rebate. Glaucoma-related healthcare costs comprise the out-of-pocket healthcare expenditure of one clinic visit at SNEC and 3 months’ worth of glaucoma medications per assessment period. When calculating glaucoma-related healthcare costs, a lower limit of SGD36.50 (the price of a subsidized doctor visit at SNEC) and an upper limit of SGD240 will be applied for those patients who spend less or exceed these limits. This means that those who qualify for a 50 % rebate will receive a rebate amount comprised between SGD18.25 and SGD120 per assessment period. Note that the rebate amounts will be explained to the patient and written down in the participant instruction booklet by the research optometrist during the baseline visit. However, emphasis will be given to the rebate rate (i.e., 25 % or 50 %) in order to be consistent with a value-based insurance design. Rebate payment is made via ebanking but the amount earned is added on the medication adherence report in order to make it fully visible to the patient.

### Outcome measures

#### Primary outcome

Our primary outcome is the *mean change from baseline in percentage of adherent days at month 6*. A day will be counted as adherent when the patients take all their medication(s) within the appropriate dosing windows for the day. The percentage of adherent days during month 1 (baseline) and month 6 will be calculated for each patient and the difference in mean change between study arms will be tested. The primary outcome is directly incentivized in the VP arm and not incentivized in the UC arm. As a result, this outcome will appropriately capture the behavioural change induced by value pricing, if any.

#### Secondary outcomes

- *Monthly mean change from baseline in percentage of adherent days at months 2 to 5* in order to monitor adherence trajectories before the study end point (month 6).
- *Monthly change from baseline in the proportion of patients with percentage of adherent days greater or equal to respectively 75 % and 90 % over the study period (i.e. from month 2 to 6)*. These outcomes are of interest as they are defined according to the adherence thresholds that VP patients need to meet in order to receive their financial incentives.
- *Mean change from baseline in IOP at month 6*.
- *Mean change from baseline in GQL-15* [38] *score at month 6* as a glaucoma-specific measure of quality of life.
- *Mean change from baseline in EQ-5D-5L* [39] *score at month 6* as a generic measure of quality of life.
- *Mean cost of financial incentives at month 6 in the VP arm. Individual cost of financial incentives will be calculated using the participants’ monthly glaucoma-related healthcare costs recorded at baseline and percentage of adherent days at month 6 (i.e. primary outcome).*

#### Explanatory outcomes

- *Monthly mean change from baseline in percentage of days where all doses where taken irrespective of time at months 2 to 6.*
- Monthly mean change from baseline in percentage of doses taken within the appropriate dosing windows *at months 2 to 6*.
- Monthly mean change from baseline in percentage of doses taken irrespective of time *at months 2 to 6*.
- *Mean change from baseline in the 8-Item Morisky Medication Adherence Score* [34–36] *(MMAS) at month 6* as this score is used to identify patients with low adherence as part of the screening process. The MMAS scale has been validated by showing that it is a good predictor of anti-hypertensive pharmacy refill adherence [34] and blood pressure (BP) control [36]. The MMAS scale has been used to measure adherence in a wide variety of chronic conditions including glaucoma [40].
- *Brief Illness Perception Questionnaire* [41] *(BIPQ) score at baseline.*
- *Specific subscale of the Beliefs about Medication Questionnaire* [42] *(BMQ) score at baseline.*

### Sample size

We chose to power the study to detect differences of ten percentage points in average monthly percentage of adherent days at 6 months between the two study arms. This effect size is sufficient to measure the effects reported by a majority of financial incentive studies [30] and is in line with a recent study that also used percentage adherence measured by electronic caps as primary end point [43]. In order to detect such effect size, a total of 100 participants is needed. This calculation assumes a two-sided statistical test of difference in means with a 5 % significance level and a power of 80 %. In addition, the standard deviation of the monthly percentage of adherent days is assumed to be no greater than 16.3 %, which is the maximum standard deviation in dose-rate adherence reported by Robin and colleagues [44]. Finally, attrition over the 6-month study is assumed to be no greater than 20 %.

### Randomization

**Fig. 2:**
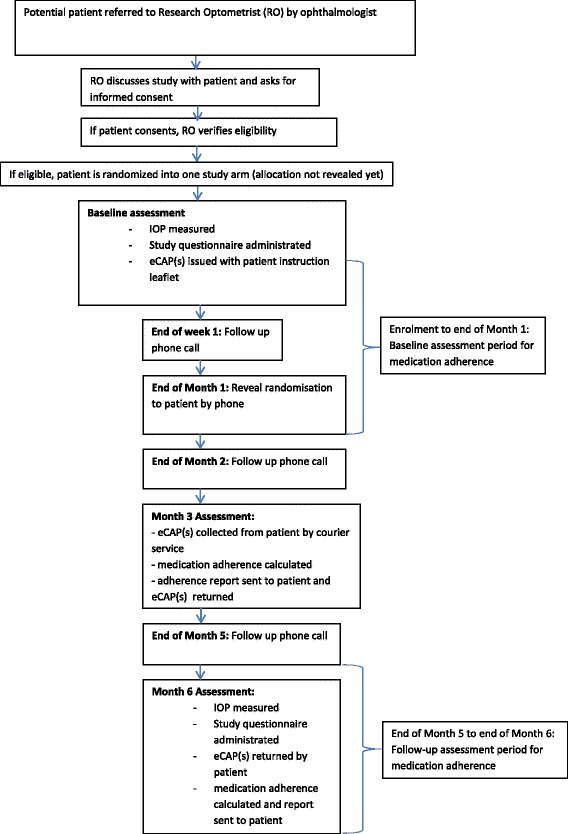
SIGMA patient timeline

A computerized random number generator (sealed envelope™) was used by the study principal investigator (PI) and coordinator at Duke-NUS to create an assignment schedule to allocate eligible participants into one of the two study arms in ratio of 1:1. Randomization was stratified according to the number of glaucoma medications prescribed to patients (one versus multiple eye drops) given that medication adherence has been shown to be highly correlated with this factor [44]. For allocation concealment, the study PI and project coordinator enclosed the assignments in sequentially numbered, opaque, sealed randomization envelopes before handing them to the research optometrist on site. Blocking size will not be disclosed to the study site to protect concealment. Note that the behavioural nature of the intervention precludes masking the arm allocation to both the study team and patient. Arm allocation will be revealed at the end of the 1-month baseline measurement of medication adherence (see Fig. 2).

### Patient recruitment, retention, withdrawal, and discontinuation

During routine clinic visits at SNEC, ophthalmologists will identify patients with problems adhering to their glaucoma eye drops and check that their GSS score does not exceed 4. The ophthalmologists will then refer the patients to a trained research optometrist to verify eligibility, take informed consent, and proceed with enrolment. Trained site research coordinators will also assist with consent taking and administer study questionnaires when required. Referral can also occur through nurse counsellors at the SNEC Glaucoma Counselling Clinic (GCC) where some non-adherent patients are referred to by their ophthalmologists. Recruitment is planned to last a year. As an incentive to join the study, patients will receive a SGD10 voucher upon successful enrolment.

Patient retention in the trial will be promoted by financial incentives. Patients will receive SGD10 when they join the study, they will also receive SGD20 for returning their eCAP(s)™ at month 3, and SGD30 at the end of the study for completing the month 6 assessment and returning the eCAP(s)™. Patients will also be engaged by means of a short adherence report they will receive at month 3 and month 6. Patient burden will be minimized by the use of a courier service to collect and return the eCAP™ in lieu of a clinic visit for the month 3 medication adherence assessment. Lost devices or broken devices will be replaced free of charge to enable the patient to continue participating in the study.

Patients are free to withdraw from the study at any time. When possible, the research optometrist will administer a short exit questionnaire to inquire about the reasons of the withdrawal and collect the study devices from the patient. Patients who withdraw will receive SGD15 for returning their eCAP(s)™ and SGD15 if they agree to complete a short exit questionnaire. In addition, patients will be discontinued from the study when their glaucoma condition deteriorates to the point of requiring surgical intervention or when they develop significant comorbid conditions that lead their ophthalmologists to discontinue their topical medications or prevents application of medications without assistance.

### Data collection

In order to record medication adherence, each patient will be issued with up to three eCAPs™ depending on the number of glaucoma medications the patient is prescribed. Each device will be labelled with a number, based on when the study device is set up and will run in ascending numerical order (the number will not relate to the patient’s unique ID number). Each vial will be labelled with the name of the patient and the medication. For patients with multiple medications the research optometrist will explain to the patient which eCAP™ number corresponds to which medication. This will be written into the patient instruction booklet (see Additional file 1) for ease of reference for patients. The research optometrist will explain to the patient how to use the eCAP™ and test that the device is properly working before the patient leaves the clinic. The patient instruction booklet also provides information on eCAP™ use along with the contact information for the study team at SNEC and Duke-NUS in case patients have any questions about their device. Patients will also be reminded to report any loss or malfunction of their devices to minimize data loss. If the patients report any issue with their devices, a replacement will be immediately arranged by courier service. In addition to receiving calls to reveal randomization and arrange device collection and delivery, the patients will receive three follow-up calls (see patient timeline, Fig. 2). During all calls, proper use and working condition of the devices will be checked. For multiple medication users, it will also be verified that the correct medication is stored in the correct vial. For further quality assurance, a copy of the eye drop dosing schedule and instructions on eCAP™ use will be sent along with the eCAPs™ and brief medication adherence report after the month 3 assessment.

IOP will be measured using Goldmann Applanation Tonometry (GAT). At baseline, the ophthalmologist will take the IOP at the point of referral. The research optometrist will then record this measurement in the baseline checklist. The same workflow will be followed at month 6 unless it is not possible to make this assessment coincide with the patient’s clinic visit. In such event, the research optometrist will take down the IOP that was taken closest to the month 6 visit from the patient’s medical record.

Paper-based questionnaires will be used at baseline and month 6. Both questionnaires include the GQL-15 and EQ-5D-5 L instruments. The baseline questionnaire also records the BIPQ and BMQ instruments along with patient socioeconomic characteristics. The month 6 questionnaire also includes the patient’s MMAS responses and score. These questionnaires will be self-administered unless the patient has difficulties in which case it will be administered by the research optometrist.

In addition, the research optometrist will fill out paper-based checklists at baseline and month 6. In addition to the patient’s IOP, the baseline checklist will record the patient’s medication regime extracted from medical records. The month 6 checklist will record the patient’s understanding and self-reported compliance to the study. The paper-based screener will also contain the self-reported MMAS responses and score.

All study documents are available in English and Mandarin to ensure the study is open to a wide range of the patient population. The research optometrist will check for completeness of all study documents and questionnaires on site and the Duke-NUS study team will double-check this at the data entry point and follow up in the event of any missing data. In efforts to promote compliance to the study requirements, all patients will receive a small monetary reward for providing key study data, as previously described - SGD10 upon successful enrollment, SGD20 for returning the eCAP™ at month 3 and SGD30 for completing the month 6 assessment and returning the eCAP™.

### Data management and monitoring

The study devices are received from patients at months 3 and 6. The project coordinator will scan each study device using the CertiScan™ desktop reader and the data will be downloaded to the Med-ic™ software. From the software the data will be exported to an Excel file where the project coordinator will check the validity of the data. She will notably check whether multiple medication users have kept their medication in the correct study device and will remedy the situation whenever possible. The project coordinator will then save the data as a text file and a statistician will run the file through a tailored Stata program that automates adherence calculation. For data that is collected via questionnaires and checklists, the project coordinator will oversee data entry at SNEC. Data will be keyed into separate Excel files that will be merged using the participant IDs. The data entry sheets have been designed to only accept values that are within the correct range, and data will be double-entered by two different study team members to minimize errors.

During the trial all paper-based documents and used study devices will be stored in locked cabinets at SNEC and Duke-NUS. After study completion, all paper-based documents will be stored in a secured room at Duke-NUS in a digital format (scanned documents stored on CD-ROM). All source documents will be retained until at least 6 months after the end of the study and then securely destroyed. Digital records will be kept for at least 15 years and securely destroyed upon the publication of all pertinent research studies/reports.

Once the first 25, 50, and 75 participants have successfully completed their month 6 assessment, primary and secondary outcomes will be computed in both study groups by a trained statistician. Attrition and missing data patterns will also be analysed. The purpose of these interim analyses is to detect potential issues that might have arisen during the data collection process before the end of the trial and to report preliminary results to SingHealth CIRB (Centralised Institutional Review Board) on an annual basis. No stopping rules have been defined for this trial.

The research optometrist will ask patients and caregivers about potential adverse events. If a patient has been hospitalized the research optometrist will copy and file the patient discharge summary if it is available. Reporting of adverse events involves notifying the SingHealth CIRB and submitting the serious adverse event (SAE) Reporting Form within the stipulated time frame. The notifying and reporting requirements depend on the severity, nature and causality of the event and there are specific procedures that must be followed [45].

No data monitoring committee will be utilized for this trial as the intervention has no impact on the standard of care received by SNEC glaucoma patients and therefore does not involve more than minimal risks. This trial is subject to study review visits of and/or audits by the SingHealth Research Quality Assurance unit, which is responsible for ensuring that all investigator-initiated research processes are conducted suitably, adequately, effectively, and efficiently across the SingHealth cluster. These study review visits/audits may be conducted routinely, triggered by CIRB or upon an investigator’s request.

### Statistical methods

#### Primary analysis

Change from baseline in percentage of adherent days at month 6 will be linearly regressed on the (i) study arm (VP vs. UC), (ii) baseline percentage of adherent days, (iii) change in number of daily doses taken in order to correct for potential medication change or number of eCAPs™ tracked, and (iv) other baseline characteristics (age, language spoken, income, IOP, BIPQ and BMQ scores) in order to increase statistical efficiency. The linear model will be estimated via ordinary least squares (OLS) and the hypothesis of equality between the mean change in primary outcome between the study arms will be tested by testing whether the binary variable indicating the study arms equals zero using a *t* test. All tests will be bilateral, performed at the 5 % level of statistical significance, and carried out using the statistical software Stata version 13.1 (StataCorp LP, College Station, TX, USA).

The analysis will be performed according to an intention-to-treat approach. If multiple medication users have faulty devices, adherence will be calculated based on functioning devices. Remaining missing adherence data will be imputed using Markov chain Monte Carlo multiple imputation including as predictors all factors mentioned above. In addition, for sensitivity analysis, baseline adherence will be carried forward for patients who withdraw from the study as those VP patients who do not achieve their goals might disproportionally withdraw from the study.

#### Secondary analyses

The same analysis described above will be conducted for the change from baseline in percentage of adherent days from months 2 to 6 in order to analyse the trajectory of adherence change induced by the intervention. All months will be simultaneously analysed and standard errors will be adjusted for individual clustering. Next, logistic regressions of indicator variables indicating whether the 75 % and 90 % adherence goals are achieved will be estimated from months 2 to 6. Regressors will include the study arm, a variable indicating whether the adherence goal was achieved at baseline, change in number of daily doses taken and the same baseline characteristics as for the primary analysis.

Changes from baseline in IOP and GQL-15 and EQ5D-5 L scores at month 6 will be linearly regressed on the study arm, their baseline values, and the same other baseline characteristics as for the primary analysis. If the intervention shows improvements (relative to the control group) both in terms of GQL-15 and EQ5D-5 L scores, we will conduct an economic analysis using utilities derived from the change in EQ5D-5 L score. The costs will be obtained from the financial incentives paid during the last month of the trial and from assumptions on operational and administrative costs incurred if the intervention were to be scaled up. The perspective of the analysis will be that of a governmental agency subsidizing healthcare costs.

#### Explanatory analyses

The same analysis as for the primary outcome will be performed using the alternative measures of medication adherence listed in the explanatory outcomes section. Next, the models used in the primary and secondary analyses will be extended by adding interaction terms between potential intervention moderators (presence of comorbidities, glaucoma- and non-glaucoma-related medication regimen, potential incentive amount, BIPQ and BMQ variables, and patient sociodemographic characteristics) and the binary variable indicating study arms. Last, medication adherence levels at baseline will be linearly regressed on potential factors of medication adherence (presence of comorbidities, glaucoma- and non-glaucoma-related medication regimen, BIPQ and BMQ variables, and patient sociodemographic characteristics).

### Ethics and dissemination

This study has been approved by the SingHealth Centralised Institutional Review Board A (Ref 2013/852/A) which oversees ophthalmology research at SingHealth. The principle investigator is responsible for informing the CIRB of any amendments to the protocol or other study-related documents, as per local requirement.

Written informed consent will be collected from each participant prior to inclusion in the study. The consent process will be carried out at SNEC. Interested and potentially eligible patients will be referred for the study by their ophthalmologist, and a trained research optometrist will explain the study to the patient in either English or Mandarin. The participant information sheet and consent form (see Additional files 4 and 5), and all other study documents will be available in both English and Mandarin. If the patient is illiterate, the research optometrist will read (or have the patient’s caregiver read) the participant information sheet and consent form in the presence of an impartial witness. In case the patient is not able to sign, the research optometrist will accept a thumb print in lieu of a handwritten signature. However, as this study will only include patients who are able to adhere to a medication regimen without assistance, patients are expected to be able to give consent to participate in the study on their own. Therefore, we did not make any additional provision for illiterate participants. Instead, we took measures to make participation as simple as possible for all patients. The research optometrist will paste the medicine label on each eCAP™ and place the eye drop(s) into the eCAP(s)™ for all participants who buy their medications on the day of recruitment. Further, eCAP™ use does not require literacy as participants only need to correctly close the eCAP™ on the medication container. This will be demonstrated by the research optometrist during the baseline visit and can be later verified by the participants as the eCAP™ beeps when correctly closed. In addition, the research coordinator will verify that all participants with multiple eye drops keep their medications in the assigned eCAP™ during the months 2 and 5 follow-up calls. No provisions have been made for informed consent to be taken from a legally acceptable representative of the patient. No provisions have been made to compensate participants for research-related injuries as the study does not involve more than minimal risks. However, compensation may be considered on a case-by-case basis for unexpected injuries due to non-negligent causes.

A unique participant ID will be assigned to all patients who are successfully enrolled on the study. All study questionnaires and checklists will only refer to the participants using this ID number so the data is de-identified. The participant patient identity log will be kept separate from all other data collected at SNEC. The hard copy will be kept in a locked cupboard with restricted access at SNEC. An electronic copy will be stored in Excel format at SNEC and will be password protected. All study materials will be kept in locked files at SNEC and Duke-NUS. Only the investigators and authorized personnel directly involved with the study will have access to the data. All data files will be password protected and stored on a secure server at Duke-NUS.

Investigators will have unrestricted access to the research data upon completion of the trial. Main trial results will be published irrespective of the magnitude and direction of the effects and their statistical significance.

## Discussion

A key consideration in incentive studies is that participants fully understand their adherence goals and reward schemes. It is in particular crucial that participants do not confuse the non-contingent payment with financial incentives as the former is given to control group participants irrespective of medication adherence while the latter is earned by intervention group participants if they reach their adherence goals. In order to make sure that this distinction has been properly understood, study team members will ask the participants during the follow-up calls to briefly describe the financial rewards they can receive and will correct any misunderstanding. The distinction between control and intervention group payments will be further emphasized in the adherence reports that will be sent at month 3 where the payment rationale is briefly re-explained. At the follow-up study visit, the understanding of the participants will be re-assessed in order to assess the quality of the study.

Further, adherence goals have been kept as simple as possible to maximize the chances that they are properly understood by all participants. This is why we defined adherent days, which are the days where all medications are taken during the appropriate time windows. The understanding of the adherence goals essentially boils down to the awareness of the medication schedule. As the medication schedule is part of usual care, no additional burden is added by the study. As some patients might not be fully aware of their medication schedule in practice, this is repeated in the patient leaflet (see Additional file 1) which is distributed to all participants at the beginning of the study. Most importantly, requiring that all doses are taken at the right time for a day to be counted as adherent emphasizes the importance of not only reducing average IOP but also to contain detrimental IOP variations.

The percentage of adherent days is then used both to define adherence goals for the participants and to calculate the primary outcome for the study. This reflects the behavioural nature of the intervention. The 5-month intervention is expected to be long enough to generate potential sustainable changes in medication taking behaviour and resulting IOP but is likely insufficient to detect meaningful differences in disease progression.

Measurement of the primary outcome critically depends on all patients keeping their medication(s) stored in their tracking device(s). This study requirement will be emphasized at recruitment and reiterated during the follow-up calls. The possibility of gaming (i.e. opening and closing the tracking device without effectively taking the medication) has also been considered. While the medication causes little side effects, some patients might want to save on medication costs. In efforts to counter and detect this potential behaviour, participants will be asked to sign a participation oath [46] and their medication purchases will be monitored. During the baseline visit, participants will be asked to keep all their medication bills and bring them to the month 6 visit. Based on the bills and a discussion with the participant, the research optometrist will assess whether the purchases correspond to 6 months of medications. While these measures are not fully fail proof, our strategy is to maximize the chances of receiving unbiased feedback by having this discussion take place after all study payments have been made to the participant and by asking nonthreatening questions on medication purchasing habits during the trial. We apply a similar strategy to verify adherence to the monitoring devices with the research optometrist asking nonthreatening questions on eCAP™ use at the end of the month 6 assessment once all payments have been made to the participant.

We were mindful of keeping the patient burden low throughout the trial. This is why we prioritized recording intervention outcomes (medication adherence, IOP, quality of life. and costs), potential reasons for non-adherence (BIPQ and BMP) and additional information at month 6 aimed at verifying data integrity as described above. Doing so, we left aside potentially interesting questions relating to medication adherence such as information on the use of digital technology that can assist patients to take their medications. We also did not track use of non-glaucoma-related medications for participants with comorbidities. However, we will be able to test whether those participants taking other medications benefit differently from the intervention. To do so, we will analyse the potential moderating effect of comorbidities and other medication regimen on glaucoma-related medication adherence and both glaucoma-related and generic quality of life. Even though there is scarce evidence on the long-term effect of continuous financial incentives [47], two recent studies, one on antipsychotic depot medication [48] and one on statins [49] showed sustained effects at one year. Our 6-month study will determine whether adherent-contingent rebates can be effective at improving medication adherence among glaucoma patients. If effective, a longer study (at least 2 years) should be undertaken to assess health gains in terms of reduction in visual field loss and long-term sustainability of medication adherence. Further, improvements in medication adherence tend to dissipate after discontinuation of the financial incentives [30]. An exception is the above study [49] that showed evidence of habit formation where adherence levels remained higher than at baseline 3 months after the incentives were discontinued. On the other hand, no study reported decreases in medication adherence below baseline levels. More generally, there is no evidence in the literature that incentives can be counterproductive for health-related behaviours [50].

The above discussion illustrates the challenges raised by the assessment of the value pricing strategy we propose. This trial will provide a rigorous assessment of this intervention that includes both mitigation strategies addressing the limitations of the design and indicators of the overall quality of the study. This study is the first to use financial incentives to increase medication adherence among glaucoma patients. More importantly, this study also uses a new form of financial incentives—adherence-contingent rebates—and it will be valuable to determine how this new approach is able to generate behavioural change. We will compare the effect of our intervention to those of other interventions aiming at improving medication adherence of glaucoma patients [51] and to those of financial incentive studies conducted in other disease areas [30].

## Trial status

Recruitment to the SIGMA study began in November 2014 and is still ongoing. As of 26 January 2016, 93 patients have been enrolled in the study.

## Abbreviations

BIPQ, Brief Illness Perception Questionnaire; BMQ, Beliefs about Medication Questionnaire; CIRB, Centralised Institutional Review Board; EQ-5D-5 L, European Quality of Life-5 Dimensions-5 Levels; GQL-15, Glaucoma Quality of Life-15 items; GSS, Glaucoma Staging System; IOP, intraocular pressure; MMAS, Morisky Medication Adherence Scale; PI, principal investigator; SAE, serious adverse events; SERI, Singapore Eye Research Institute; SIGMA, Study on Incentives for Glaucoma Medications Adherence; SNEC, Singapore National Eye Centre; UC, usual care; VP, value pricing.

## Additional files

Additional file 1: SIGMA Participant Instruction Booklet. This booklet is given to all patients who join the study. The booklet contains study information and study team contact details. Additional details are added to reflect the individual patient’s dosing schedule, 3-month healthcare costs and rebate amounts. (PDF 844 kb) Additional file 2: Participant Medication Adherence Report A_month 3 (usual care arm). This report is sent to patients in the usual care arm after month 3 to inform patients of their adherence and explain the non-contingent payment. (PDF 205 kb) Additional file 3: Participant Medication Adherence Report A_month 3 (value pricing arm). This report is sent to patients in the value pricing arm after month 3 to inform patients of their adherence and explain the rebate amount they have earned. (PDF 207 kb) Additional file 4: Participant Information Sheet and Consent Form (English). All English-speaking patients will complete this consent form before joining the study. The patient signs two copies, one copy is kept by SERI, and one copy is given to the patient. (PDF 298 kb) Additional file 5: Participant Information Sheet and Consent Form (Mandarin). All Mandarin-speaking patients will complete this consent form before joining the study. The patient signs two copies, one copy is kept by SERI, and one copy is given to the patient. (PDF 252 kb)
