# Supplementary material for: Study on Incentives for Glaucoma Medication Adherence (SIGMA): study protocol for a randomized controlled trial to increase glaucoma medication adherence using value pricing
Source: Trials. 2016 Jul 15;17:316. doi: 10.1186/s13063-016-1459-1 (PMC4947326; doi:10.1186/s13063-016-1459-1)
Supplement: Additional file 1: — SIGMA Participant Instruction Booklet. This booklet is given to all patients who join the study. The booklet contains study information and study team contact details. Additional details are added to reflect the individual patient’s dosing schedule, 3-month healthcare costs and rebate amounts. (PDF 844 kb) [file 13063_2016_1459_MOESM1_ESM.pdf]

SINGAPORE EYE RESEARCH INSTITUTE

DUKE NUS
GRADUATE MEDICAL SCHOOL SINGAPORE

SIGMA GLAUCOMA MEDICATION ADHERENCE STUDY

Participant Instruction Booklet

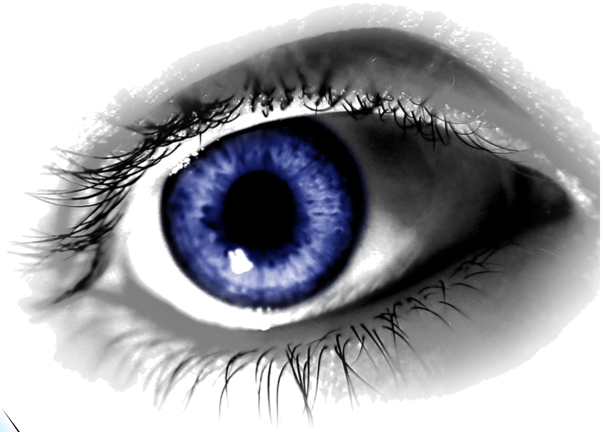

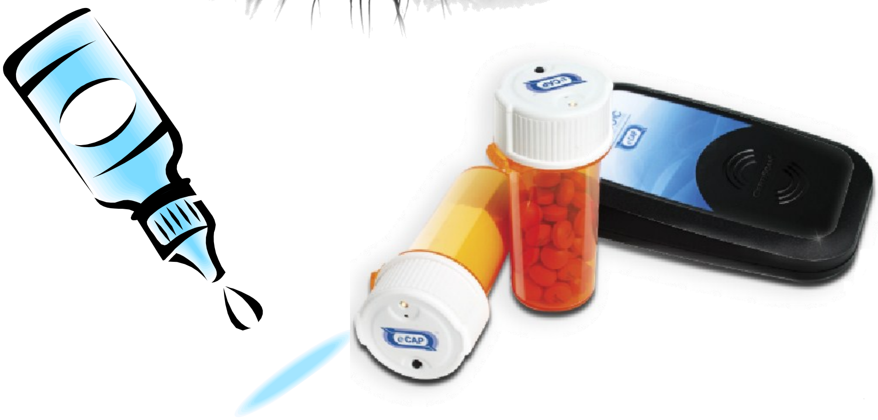

Version 2, 01 Jul 2015

For more information, please call

Or email

Section A: Study Design

Our **6-month study** seeks to test whether glaucoma patients will use **their eye drops more regularly** if provided **subsidies** for medication and doctor's visit costs

We will compare the medication adherence of participants *randomly allocated* into one of two arms:

SUBSIDY arm

Earn *subsidies* by meeting medication adherence goals

USUAL CARE arm

Receive *fairness payments* regardless of medication adherence

What would you have to do?

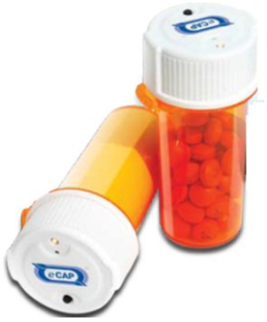

- Keep your glaucoma eye drops in eCAP containers (provided free of charge during the study) that monitor your medication use

- Only open your eCAPs when using your eye drops

- Return your eCAPs at month 3 & 6

- Attend 2 assessment sessions at SNEC

Only participants who complete all study requirements will be provided with study subsidies or reimbursements.

Section B: Instructions on eCAP Use

What is an eCAP?

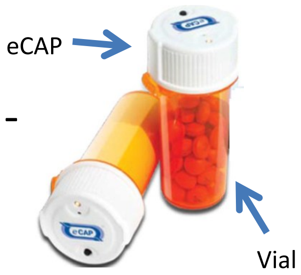

- The eCAP is a device with an in-built electronic tag which records the time whenever it is opened.

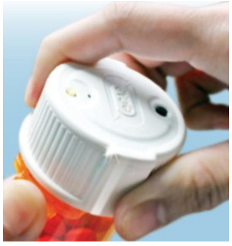

- When you open the eCAP to take out your medication, the time will be recorded.

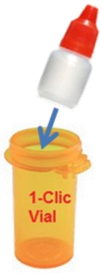

- Store your glaucoma eye drop medication inside the vial covered by the eCAP at all times.

- Only remove eCAP(s) when you are instilling your glaucoma medication. Avoid unnecessary opening of the vial, as it records each time you unscrew the eCAP.

- Use your medication within the dosing windows indicated in your **Eye Drop Dosing Schedule (Section C)**.

- When closing, twist the eCAP until you hear a “beep” sound and see a green light flash. This signals that the eCAP is locked in a proper position.

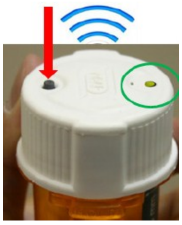

- Return eCAP(s) to study team for data extraction at month 3 and 6 visits.

Section C: Eye Drop Dosing Schedule

Please instill all eyedrop doses within the indicated dosing windows (marked with an X) to be considered adherent to your medication(s).

1	eCAP ID	Brand Name	Eye(s)<br>☐ Left<br>☐ Right<br>☐ Both
	Dose<br>Drop(s)	Frequency<br>x daily	

Dosing windows		
Morning	Afternoon	Evening

2	eCAP ID	Brand Name	Eye(s)
	Dose<br><br>Drop(s)	Frequency<br><br>x daily	☐ Left<br>☐ Right<br>☐ Both

Dosing windows		
Morning	Afternoon	Evening

3	eCAP ID	Brand Name	Eye(s)
	Dose<br><br>Drop(s)	Frequency<br><br>x daily	☐ Left<br>☐ Right<br>☐ Both

Dosing windows		
Morning	Afternoon	Evening

Section D: Instructions on Adherence Calculation

- Medication use is recorded with each eCAP opening.

- Only eCAP openings **within** the appropriate dosing windows in your **(C) Eye Drop Dosing Schedule** will contribute towards your adherence targets.

- You are considered adherent to your glaucoma medication for a day when you instill all doses for all prescribed glaucoma eye drops within the dosing windows for that particular day.

**Assessment Period**	**Monitoring Period**
Baseline Assessment	Week 1—4
Month 3 Assessment	Week 5—12
Month 6 Assessment	Week 15—27

Adherence for each period is calculated as:

$$\frac{\text{No. of adherent days}}{\text{No. of days in monitoring period}} \times 100\%$$

Reports on your medication adherence will be sent to you at month 3 and 6.

Only participants in the <u>SUBSIDY arm</u> have the opportunity to <u>earn subsidies</u>

Section E: Instructions on Subsidy Calculation

- Subsidies are applied to an estimate of your 3 month healthcare costs at the month 3 and 6 assessments.

- Consultation and medication receipts collected from you at enrolment are used to estimate your 3 month healthcare costs

Your estimated 3 month healthcare costs = ____________

Based on the proportion of days that you are adherent to your medication over each monitoring period (at month 3 & 6), you will receive a certain percentage of subsidies on your estimated 3 month healthcare costs.

Monitoring period	Total no. of days	Adherent days to reach 90% target	Adherent days to reach 75% target
1	56	51	42
2	84	76	64
Subsidies given for healthcare costs		50%	25%
Subsidies for 3 months of healthcare costs			

Section F: Study & Payment Schedule

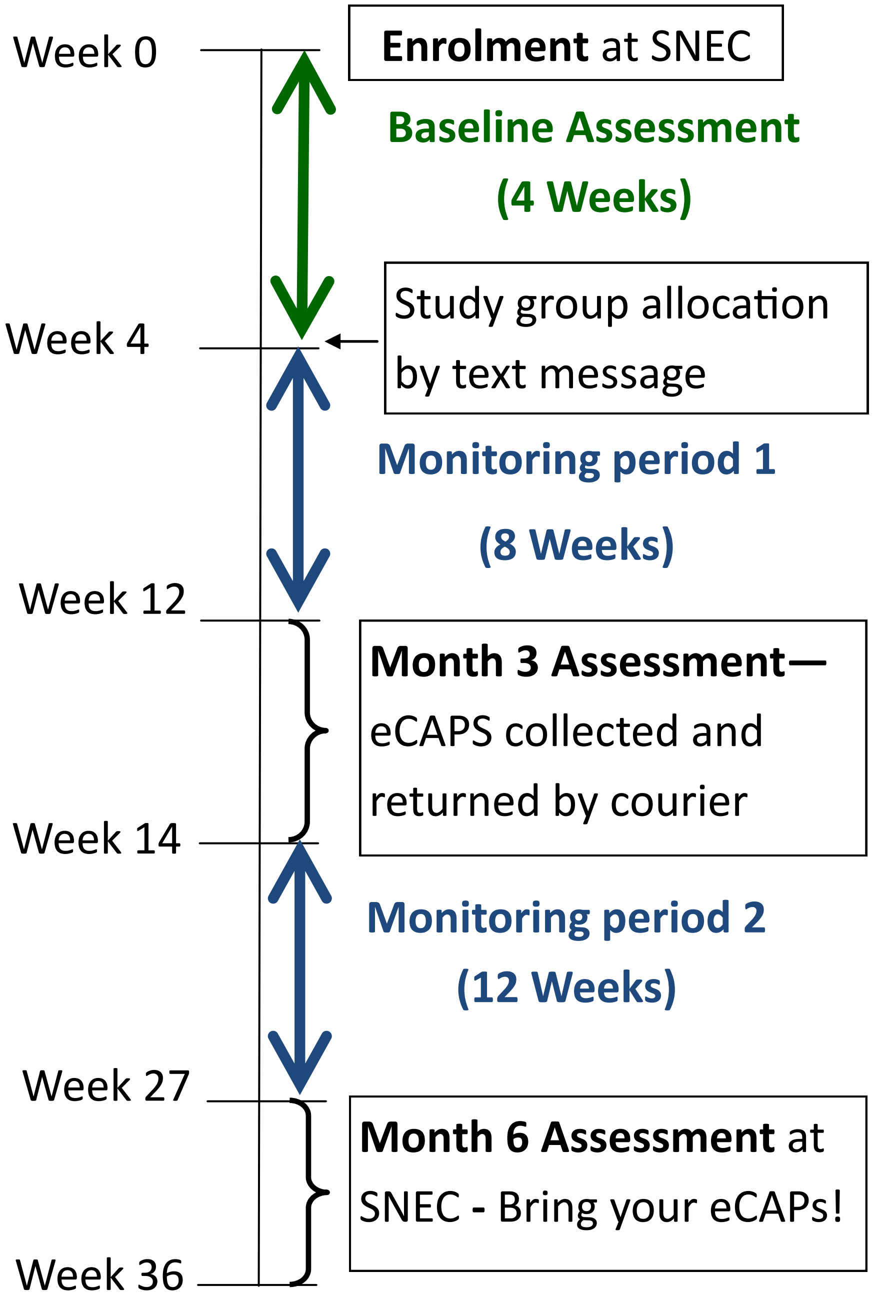

Payment Schedule		
**Week from baseline : __________**	SUBSIDY arm	USUAL CARE arm
**Enrolment (Week 0)**	$10 in NTUC Vouchers	
**Month 3 Assessment (Week 13 – 14)**	Bank transfer of $20 for return of eCAPs	
	Bank Transfer of subsidies earned	Bank Transfer of $30 *fairness payment*
**Month 6 Assessment (After Week 27)**	$30 NTUC vouchers for completion of month 6 assessment	
	Bank transfer of subsidies earned (Within 2 weeks of eCAP return)	$30 NTUC voucher *fairness payment*

Frequently asked questions

What should I do about a lost / damaged eCAP / eCAP that does not close with a "beep" sound and a flashing of a green light?

- Please get in touch with the study team who will arrange for a replacement eCAP to be sent to you free of charge

When will I receive my study payments?

- All study payments that are not issued in NTUC vouchers will be made available by bank transfer within 2 weeks of eCAP return to the study team

Study Contact Numbers

Assistance on eCAPs & study appointments:

Study Research Optometrist

[redacted]

SINGAPORE EYE RESEARCH INSTITUTE

11 Third Hospital Avenue #05-00, SNEC Building, (S) 168751

[redacted]

Assistance on adherence & subsidies:

Study Project Coordinator

[redacted]

DUKE NUS GRADUATE MEDICAL SCHOOL SINGAPORE

8 College Road, Level 4, Duke NUS Graduate Medical School, (S) 169857

[redacted]
