# Supplementary material for: Study on Incentives for Glaucoma Medication Adherence (SIGMA): study protocol for a randomized controlled trial to increase glaucoma medication adherence using value pricing
Source: Trials. 2016 Jul 15;17:316. doi: 10.1186/s13063-016-1459-1 (PMC4947326; doi:10.1186/s13063-016-1459-1)
Supplement: Additional file 2: — Participant Medication Adherence Report A_month 3 (usual care arm). This report is sent to patients in the usual care arm after month 3 to inform patients of their adherence and explain the non-contingent payment. (PDF 205 kb) [file 13063_2016_1459_MOESM2_ESM.pdf]

SIGMA STUDY PARTICIPANT MEDICATION ADHERENCE REPORT

Dear control arm participant,

Thank you for your continued participation in the SIGMA study!

We would like to remind you that control arm participants will receive a $30 fairness payment at month 3 & 6 for their use of the eCAP(s) and compliance with all other study requirements. Please remember that control arm participants do not earn subsidies for meeting medication adherence goals.

Below is a summary of your medication adherence that has been recorded by your eCAP(s) between DDMMYY and DDMMYY.

MEDICATION ADHERENCE SUMMARY FOR MONTH 2-3

**Time period**	DDMMYY - DDMMYY
**No. of days where adherence target was met**	**Proportion of days where adherence target was met**
__ out of __ days	Adherent on __% of days

Your $30 fairness payment, along with a $20 reimbursement for returning your eCAP(s), will be transferred to the bank account that you indicated in the participant oath during enrolment into this study.

Should you fail to receive the payment within two weeks of the receipt of this report, or have any questions regarding the calculation of your adherence, please contact the study coordinator [redacted] at [redacted] (8.30am and 6.00pm, Monday – Friday) or send an email to [redacted]

eCAP USE FOR MONTH 4 - 6

Please put your designated medication into the eCAP-covered vial(s) and continue with your recommended medication dosing schedule as you did during the first 3 months of the study. A participant instruction leaflet on eCAP use has been included for your reference.

Should you have any questions regarding the use of your eCAP(s) or your medication and doctor's consultation visits, please contact the study research optometrist [redacted] at [redacted] (8.30am and 6.00pm, Monday – Friday) or send an email to [redacted]

Sincerely,

SIGMA Study Team
