# Supplementary material for: Study on Incentives for Glaucoma Medication Adherence (SIGMA): study protocol for a randomized controlled trial to increase glaucoma medication adherence using value pricing
Source: Trials. 2016 Jul 15;17:316. doi: 10.1186/s13063-016-1459-1 (PMC4947326; doi:10.1186/s13063-016-1459-1)
Supplement: Additional file 3: — Participant Medication Adherence Report A_month 3 (value pricing arm). This report is sent to patients in the value pricing arm after month 3 to inform patients of their adherence and explain the rebate amount they have earned. (PDF 207 kb) [file 13063_2016_1459_MOESM3_ESM.pdf]

SINGAPORE EYE RESEARCH INSTITUTE

DUKE NUS
GRADUATE MEDICAL SCHOOL SINGAPORE

SIGMA STUDY PARTICIPANT MEDICATION ADHERENCE REPORT

Dear subsidy arm participant,

Thank you for your continued participation in the SIGMA study!

We would like to remind you that subsidy arm participants can earn subsidies by meeting medication adherence goals. Please note that subsidies will be applied to the estimated cost for 3 months of glaucoma medication and one doctor consultation at SNEC as assessed for you by the Research Optometrist during your baseline visit.

Below is a summary of your medication adherence that has been recorded by your eCAP(s) between DDMMYY and DDMMYY, and the amount of subsidies you are entitled to, based on this information.

MEDICATION ADHERENCE SUMMARY FOR MONTH 2 - 3

**Time period**	DDMMYY - DDMMYY		
**No. of days where adherence goal was met**	**Proportion of days where adherence goal was met**	**Subsidies earned for medication and consultation**	**Value of Subsidies earned**
__ out of __ days	Adherent on __% of days	__%	$__

Your subsidy, along with a $20 reimbursement for returning your eCAP(s), will be transferred to the bank account that you indicated in your participant oath during enrolment into this study.

Should you fail to receive the bank transfer within two weeks of the receipt of this report, or have any questions regarding the calculation of your subsidy, please contact the study coordinator [redacted] at [redacted] (8.30am and 6.00pm, Monday – Friday) or send an email to [redacted]

eCAP USE FOR MONTH 4 - 6

Please put your designated medication into the eCAP-covered vial(s) and continue with your recommended medication dosing schedule as you did during the first 3 months of the study. A participant instruction leaflet on eCAP use and subsidies has been included for your reference.

Should you have any questions regarding the use of your eCAP(s) or your medication and doctor's consultation visits, please contact the study research optometrist [redacted] at [redacted] (8.30am and 6.00pm, Monday – Friday) or send an email to [redacted]

Sincerely,

SIGMA Study Team
