# Supplementary material for: Study on Incentives for Glaucoma Medication Adherence (SIGMA): study protocol for a randomized controlled trial to increase glaucoma medication adherence using value pricing
Source: Trials. 2016 Jul 15;17:316. doi: 10.1186/s13063-016-1459-1 (PMC4947326; doi:10.1186/s13063-016-1459-1)
Supplement: Additional file 4: — Participant Information Sheet and Consent Form (English). All English-speaking patients will complete this consent form before joining the study. The patient signs two copies, one copy is kept by SERI, and one copy is given to the patient. (PDF 298 kb) [file 13063_2016_1459_MOESM4_ESM.pdf]

PARTICIPANT INFORMATION SHEET

You are being invited to participate in a research study.

Before you take part in this research study, the study must be explained to you and you must be given the chance to ask questions. Please read carefully the information provided here. If you agree to participate, please sign the informed consent form. You will be given a copy of this document to take home with you.

STUDY INFORMATION

Protocol Title:

A Randomized Controlled Trial to Increase Glaucoma Medication Adherence using Value Pricing

Principal Investigator:

Marcel BILGER
Assistant Professor, Health Systems and Services Research; Duke-NUS Graduate Medical School.
8 College Road Singapore 169857.
[redacted]

Site Principal Investigator:
Assoc Prof Tina Wong
Senior Consultant, Glaucoma Service
Singapore National Eye Centre

Sponsor:

National Medical Research Council (NMRC), Ministry of Health (MOH)

PURPOSE OF THE RESEARCH STUDY

You are being invited to participate in a research study of *looks at the effects of monetary rewards for the use of glaucoma eye drops in accordance with dosing regimes.* We hope to learn about the *effectiveness and cost-effectiveness of rewards for encouraging greater adherence to glaucoma medication.* You were selected as a possible subject in this study because *you fulfil the following conditions:*

i. Singaporean citizen or permanent resident
ii. Patient at the Singapore National Eye Centre (SNEC)
iii. Conversant in English or Mandarin
iv. Aged 21 to 85 years of age
v. Taking at least one glaucoma drug

This study will recruit 100 subjects from Singapore National Eye Centre (SNEC) over a period of Nov 2014 to Aug 2016. About 100 subjects will be involved in this study. Study participation is expected to last 6 months and will come alongside your regular physician and pharmacy visits at SNEC. Depending on the study arm you are assigned to, you may also be offered opportunities to earn financial rewards contingent on the use of your glaucoma medications according to the

dosing schedule agreed on with your nurse counsellor at the Glaucoma Counselling Clinic (GCC).

STUDY PROCEDURES AND VISIT SCHEDULE

If you agree to take part in this study, you will be *will be randomised* to 1 of 2 Study groups. You have a 50% chance of being randomized into Group 1 and a 50% chance of being randomized into Group 2:*

- **<u>Group 1 (Usual care - UC)</u>**: The patients in this study arm will benefit from the glaucoma counselling that is provided by SNEC to its non-adherent patients.
- **<u>Group 2 (Subsidy - S)</u>**: Participants in the S arm will receive identical treatment to those in the UC arm. In addition, they will have the chance to earn a rebate on their medications and visits for taking their medication(s) within the appropriate dosing window(s) between physician visits.

**Randomisation means assigning you to one of 2 groups by chance, like tossing a coin or rolling dice.*

<u>Your participation in the study will last *6 months*.</u> If you agree to take part in this study, you will be asked to:

- Store each of your glaucoma eye drop medications in a container covered by a medication cap (eCAP) which tracks medication use with each opening over the 6 month study period.
- Visit the doctor's office *2* times in the course of the study, once prior to enrolment and once prior to the final assessment.
- Return your eCAPs at month 3 via a courier service and in person at month 6 when you attend your final study assessment and physician visit at SNEC. In exceptional circumstances when a patient does not have a physician visit between the month 6 and month 9 study period the month 6 assessment may be completed by phone. In this case the eCAP(s) will be collected by courier and study payments will be made by bank transfer.
- Participate in surveys and measurements at the start of the study (baseline) and month 6.

<u>If you are in the subsidy arm,</u> you will be offered the following rebate on the price* of your physician visit and glaucoma medications at month 3 and 6:

- **50% subsidy** over the previous 3 months
  If you return the eCAP and it shows that you took your medication(s) within the appropriate dosing window(s) on 90% of the days between visits.

- **25% subsidy** over the previous 3 months
  If you return the eCAP and it shows that you took your medication(s) within the appropriate dosing window(s) on between 75% and 90% of the days.

- Final subsidies to be paid will be dependent on medication and physician receipts that you submit to the study Research Optometrist at enrolment and will be based on your estimated 6 month healthcare costs of up to $480.

- Subsidies earned will be transferred to a bank account of your choice.

- No subsidy will be provided to participants who do not complete the assessment and/or cannot show evidence of adherence on at least 75% of the days between visits. Only eCAP data will be counted as evidence of adherence.

**To avoid a price increase for the participants, the reference price will not be the market price*

but will include the Government subsidies that subsidized patients receive for their care.

If you are in the usual care arm, you will be offered a $30 fairness payment at each 3 month time point for your efforts to comply with use of the study eCAPs.

Schedule of visits and procedures:

Visit 1: Physician visit followed by enrolment and baseline assessment with Research Optometrist at SNEC.

Final Visit (Week 26): Physician visit followed by month 6 assessment with Research Optometrist at SNEC. In exceptional circumstances when a patient does not have a physician visit between the month 6 and month 9 study period the month 6 assessment may be completed by phone. In this case the eCAP(s) will be collected by courier and study payments will be made by bank transfer.

YOUR RESPONSIBILITIES IN THIS STUDY

If you agree to participate in this study, you should*:*

- Store your glaucoma eye drops in the study device (eCAP) over the 6 month study duration as instructed and follow the advice given to you by the study team.
- Keep your study appointments. If it is necessary to miss an appointment, please contact the study staff to reschedule as soon as you know you will miss the appointment.
- Inform the Principal Investigator as soon as possible about any side effects that you may have encountered.
- Be prepared to visit the hospital *2 times* and undergo all the procedures that are outlined above.

WITHDRAWAL FROM STUDY

You are free to withdraw your consent and discontinue your participation at any time without prejudice to you or effect on your medical care. If you decide to stop taking part in this study, you should tell the Principal Investigator.

- If you withdraw from the study, *Contact the Research Optometrist and whenever possible provide the reason for withdrawal through a brief feedback questionnaire.*
- *Return the eCAPs promptly within 30 days of notification of intent to withdraw.*

Your doctor, the Principal Investigator and/or the Sponsor of this study may stop your participation in the study at any time for one or more of the following reasons*:*

- Failure to follow the instructions of the Principal Investigator and/or study staff.
- The Principal Investigator decides that continuing your participation could be harmful.
- You need treatment not allowed in the study.
- The study is cancelled.

WHAT IS NOT STANDARD CARE OR EXPERIMENTAL IN THIS STUDY

The study is being conducted because the use of incentives is not yet proven to be a standard option in increasing medication adherence in subjects with glaucoma. We hope that your participation will help us to determine the effectiveness as well as cost-effectiveness of incentives.

Use of randomization (study arm selection by chance) is only done for research studies.

POSSIBLE RISKS, DISCOMFORTS AND INCONVENIENCES

- The use of eCAPs will pose minimal inconvenience due to its relative ease of use and portability.
- No more than minimal risk is expected as a consequence of your participation. You will receive standard care for glaucoma at SNEC.

POTENTIAL BENEFITS

If you participate in this study you may reasonably expect to benefit from the study intervention through the use of one or more strategies for improving your medication adherence and possibly your health. Additionally, your participation will contribute to the medical knowledge about the use and cost-effectiveness of these incentives on medication adherence. You may also earn money and cash vouchers for your participation.

ALTERNATIVES

If you choose not to take part in this study, the alternative is to have what is considered standard care for your condition. In our institution this would be routine check-ups with an ophthalmologist, prescriptions for glaucoma eye drops, and glaucoma counselling if you are found to be non-adherent to your glaucoma eye drops by your opthalmologist.

Glaucoma counselling has the following potential benefits:
Patients learn about effective glaucoma treatment, including: understanding glaucoma and visual field process, notably risk factors and symptoms; management and treatment of glaucoma; medications and usage such as tips on self-application of eye drops. In addition, a nurse counsellor will also discuss optimal dosage windows for each medication with special emphasis on the risks of non-adherence to the treatment and help the patient come up with a dosing schedule that compliments the patient's lifestyle, taking into consideration her working hours.

SUBJECT’S RIGHTS

Your participation in this study is entirely voluntary. Your questions will be answered clearly and to your satisfaction.

In the event of any new information becoming available that may be relevant to your willingness to continue in this study, you or your legal representative will be informed in a timely manner by the Principal Investigator or his/her representative.

By signing and participating in the study, you do not waive any of your legal rights to revoke your consent and withdraw from the study at any time.

CONFIDENTIALITY OF STUDY AND MEDICAL RECORDS

Information collected for this study will be kept confidential. Your records, to the extent of the applicable laws and regulations, will not be made publicly available. Only your Investigator(s) and study team (Both Duke NUS and SERI) will have access to the confidential information being collected.

However, the Regulatory Agencies, Institutional Review Board and Ministry of Health will be granted direct access to your original medical records to check study procedures and data, without making any of your information public.

By signing the Informed Consent Form attached, you or your legal representative are authorizing (i) collection, access to, use and storage of your “Personal Data, and (ii) disclosure to authorised

service providers and relevant third parties.

“Personal Data” means data about you which makes you identifiable (i) from such data or (ii) from that data and other information which an organisation has or likely to have access. This includes medical conditions, medications, investigations and treatment history.

Research arising in the future, based on this Personal Data, will be subject to review by the relevant institutional review board.

By participating in this research study, you are confirming that you have read, understood and consent to the SingHealth Data Protection Policy- the full version is available at www.singhealth.com.sg/pdpa. Hard copies are also available on request.

Data collected and entered into the Data Collection Form(s) are the property of *Duke-NUS Graduate Medical School*. In the event of any publication regarding this study, your identity will remain confidential.

COSTS OF PARTICIPATION

If you take part in this study, the following will be performed at no charge to you: *Provision and replacement of eCAPs for the study*

If you take part in this study, you will have to pay for the following: *All routine physician visits and glaucoma medication that you purchase during the 6 month study period*

You will be reimbursed for your time, inconvenience and transportation costs as follows:

- If you complete the study, you will be paid $60.
- If you do not complete the study for any reason, you will be paid:
  - $10 for successful enrolment in the study and completion of the baseline assessment survey
  - $20 for returning your eCAP(s) at month 3
  - $30 for completing the final assessment at month 6, returning your eCAP(s) and completing your month 6 assessment survey.

RESEARCH RELATED INJURY AND COMPENSATION

The Hospital does not make any provisions to compensate study subjects for research related injury. However, compensation may be considered on a case-by-case basis for unexpected injuries due to non-negligent causes.

By signing this consent form, you will not waive any of your legal rights or release the parties involved in this study from liability for negligence.

WHO TO CONTACT IF YOU HAVE QUESTIONS

If you have questions about this research study or in the case of any injuries during the course of this study, you may contact the Principal Investigator Marcel Bilger [redacted] or the study coordinator [redacted] at [redacted] during office hours (8.30am to 6.00pm).

If you have questions about the study or your rights as a participant, you can call the SingHealth Centralised Institutional Review Board, which is the committee that reviewed and approved this study, the telephone number is [redacted] during office hours (8:30 am to 5:30pm).

This study has been reviewed by the SingHealth Centralised Institutional Review Board for ethics approval.

If you have questions about your rights as a participant, you can call the SingHealth Centralised Institutional Review Board at [redacted] during office hours (8:30 am to 5:30pm).

If you have any complaints about this research study, you may contact the Principal Investigator or the SingHealth Centralised Institutional Review Board.

CONSENT BY RESEARCH SUBJECT

Details of Research Study

Protocol Title:

A Randomized Study on Economic Incentives for Glaucoma Medications Adherence (“SIGMA”)

Principal Investigator:
Dr Marcel Bilger

Site Principal Investigator:
Assoc Prof Tina Wong

Subject’s Particulars

Name: NRIC No.:

Address:

Sex: Female/Male Date of birth _______________
dd/mm/yyyy

Race: Chinese/ Malay/ Indian /Others (please specify) ________________________

I, _____________________________________(NRIC/Passport No.________________________)
(Name of patient)
agree to participate in the research study as described and on the terms set out in the Patient Information Sheet.

I have fully discussed and understood the purpose and procedures of this study. I have been given the Participant Information Sheet and the opportunity to ask questions about this study and have received satisfactory answers and information.

I understand that my participation is voluntary and that I am free to withdraw at any time, without giving any reasons and without my medical care being affected.

By participating in this research study, I confirm that I have read, understood and consent to the SingHealth Data Protection Policy. I also consent to the use of my Personal Data for the purposes of engaging in related research arising in the future.

_______________________________________ ______________________
Signature/Thumbprint (Right / Left) of participant Date of signing

To be filled by parent / legal guardian / legal representative, where applicable

I, ___________________________ hereby give consent for the above participant to participate in
(parent / legal guardian)
the proposed research study. The nature, risks and benefits of the study have been explained clearly to me and I fully understand them.

____ ________________________
Signature/Thumbprint (Right / Left) of parent /legal guardian Date of signing

Translator Information (if required)

The study has been explained to the participant/ legal representative in

____________________________________by ____________________________________.
Language Name of translator

To be filled witness, where applicable

An impartial witness should be present during the entire informed consent discussion if a subject or the subject's legal representative is unable to read. After the written informed consent form and any written information to be provided to subjects, is read and explained to the subject or the subject's legal representative, and after the subject or the subject's legal representative has orally consented to the subject's participation in the study and, if capable of doing so, has signed and personally dated the consent form, the witness should sign and personally date the consent form.

Witnessed by: ________________________________ ________________________
Name of witness Designation of witness

________________________________ ________________________
Signature of witness Date of signing

Investigator's Statement

I, the undersigned, certify to the best of my knowledge that the patient/patient's legal representative signing this informed consent form had the study fully explained and clearly understands the nature, risks and benefits of his/her / his ward's / her ward's participation in the study.

________________________ ________________________ ________________
Name of Investigator Signature Date
