# Supplementary material for: Study on Incentives for Glaucoma Medication Adherence (SIGMA): study protocol for a randomized controlled trial to increase glaucoma medication adherence using value pricing
Source: Trials. 2016 Jul 15;17:316. doi: 10.1186/s13063-016-1459-1 (PMC4947326; doi:10.1186/s13063-016-1459-1)
Supplement: Additional file 5: — Participant Information Sheet and Consent Form (Mandarin). All Mandarin-speaking patients will complete this consent form before joining the study. The patient signs two copies, one copy is kept by SERI, and one copy is given to the patient. (PDF 252 kb) [file 13063_2016_1459_MOESM5_ESM.pdf]

SingHealth

参与者信息表

在此，您被邀请参与一项研究。

在您决定是否参与本研究前， 我们将向您详细介绍本研究，并且将给予您提出疑问的机会。请认真阅读这份资料。若您愿意参与本研究,请您在知情同意书上签字。您将会得到一份此文件。

研究资料

研究名称:一项随机对照研究，研究以奖励是否能鼓励更好地按照剂量点滴青光眼药水

A Randomized Controlled Trial to Increase Glaucoma Medication Adherence using Value Pricing

研究总负责人：
Marcel BILGER
助理教授，卫生系统与服务研究部; 杜克-新加坡国大医学研究生院.
地址: 8 College Road Singapore 169857
电话: [redacted]

研究分支总负责人
Tina Wong 副教授
高级顾问，青光眼部门
新加坡全国眼科中心

资助机构: 新加坡国家医学研究理事会(NMRC)，新加坡卫生部(MOH)

研究目的

您被邀请参与一项研究金钱奖励对于按照剂量点滴青光眼药水的影响。本研究结果，预期能告知以奖励鼓励更好地按照剂量点滴青光眼药水的有效性及成本效益。您之所以被选为本研究适合的招募对象是因为您符合了以下所有的标准：

i. 新加坡公民或永久居民
ii. 在新加坡全国眼科中心看诊
iii. 能以英语或华语对话
iv. 年龄介于 21 至 85 岁
v. 至少使用一种青光眼药物

本研究将于 2014 年 11 月至 2016 年 8 月间从新加坡全国眼科中心招募 100 位参与者，本研究将涉及约 100 名参与者。本研究预计将持续 6 个月，研究复诊日期，将配合您于新加坡全国眼科中心的看诊及取药同日进行。根据您分配到的研究组，您将有机会以按照您与青光眼辅导诊所的护士同意的剂量点滴青光眼药水的方式，赚取金钱奖励。

研究程序及检查时间表

若您同意参与本研究，您将随机*分配至第一或第二研究组，您被分配至第一或第二研究组的几率各占一半：

- **第一组（常规护理- UC）：** 分配至此研究组的参与者将受益于新加坡全国眼科中心为不遵守用药的病患，所提供的青光眼辅导。

- **第二组(津贴组- S)：** 分配至津贴组的参与者，除了会接受与常规护理相同的治疗外，若您在此次看诊与下次复诊期间定时使用药物，您将有机会赚取药物及看诊津贴。

*随机是以偶然的机会将您分配至第一或第二研究组，如投硬币或掷骰子。

您于本研究的参与将持续大约 6 个月。若您同意参与本研究，我们将要求您：

- 将您的青光眼药物放置在电子容器（eCAPs）内，以记录您接下来 6 个月的药物使用
- 到新加坡全国眼科中心进行两次看诊，基线检查及第 6 个月看诊
- 在第 3 个月时将电子容器（eCAPs）归还给我们的快递员及在第 6 个月出席第 6 个月看诊并亲自归还电子容器（eCAPs）。在特殊情况下，如果参与者在第 6 个月和 9 个月之间没有医生访问时，第 6 个月的评估可以通过电话完成。在这种情况下快递员会收回（eCAPs）而研究补贴将会通过银行转账。
- 完成基线及第 6 个月的问卷调查及测试

若您备份配至津贴组，以下列明我们将给予您在第 3 及第 6 个月药物及看诊津贴*：

- 为过去的三个月的药物给予 **50% 的津贴**
  若您归还电子容器（eCAPs），电子容器（eCAPs）显示您 90%的时间定时使用药物。

- 为过去的三个月的药物给予 **25% 的津贴**
  若您归还电子容器（eCAPs），电子容器（eCAPs）显示您 75%至 90%的时间定时使用药物。

- 我们将根据您基线检查时提交给研究人员的药物及看诊收据，估计您 6 个月的医疗花费（顶限为＄480），从而计算您所能得到的津贴。

- 您所获得的津贴将转帐进您所选择的银行户口。

- 若您不完成问卷调查及测试，或无法证明您至少 75%的时间定时使用药物，您将不会获得任何津贴。只有电子容器（eCAPs）记录将被视为定时使用药物的证明。

*为了避免参与者付更高的价钱，参考价将包括所有的政府补贴，而非市价。

若您备份配至常规护理组，我们会为因您遵守使用电子容器（eCAPs），而提供＄30 的“公平补贴”。

研究程序及检查时间表：

看诊 1 ： 例常检查后由研究人员于新加坡全国眼科中心进行筛选及基线测试

最后一次看诊(第 26 个星期)：例常检查后由研究人员于新加坡全国眼科中心进行第 6 个月测试）。在特殊情况下，如果参与者在第 6 个月和 9 个月之间没有医生访问时，第 6 个月的评估

可以通过电话完成。在这种情况下快递员会收回（eCAPs）而研究补贴将会通过银行转账。

您在本研究中的责任

若您同意参与本研究，您应当：

- 在参与研究的 6 个月内将您的青光眼药水放置在电子容器（eCAPs）内，并听从研究人员的指示与建议。
- 按时出席研究预约，若需重新安排您的预约，请尽快联系研究人员重新安排。
- 若您遇到任何副作用或不良反应时，请尽快通知研究总负责人。
- 请准备到医院 2 次，以进行上述的程序

退出研究

在不对您引起偏见，不影响您的医疗服务的情况下，您可在研究过程中随时撤消同意并退出研究。若您决定退出研究, 请您通知研究总负责人。

- 若您决定退出研究，请通知研究人员，并尽可能完成一份简单的问卷以提供退出的原因。
- 在通知退出研究的 30 天内尽快退还电子容器（eCAPs）

您的医生、研究总负责人及/或研究赞助商，有权因以下一或多种原因，在任何时间中止您在本研究中的参与：

- 您未能遵循医生或研究人员的指令。
- 研究医生认为继续研究可能带来害处。
- 您需要进行本研究中不被允许的疗程
- 本研究被中断

研究里非标准医疗照顾或属实验性质事项:

本研究的进行是因为以奖励鼓励更好地按照剂量点滴青光眼药水还未被确定是否有效。您的参与将有助于我们确定其有效性及成本效益。

随机分配（以偶然的机会分配研究组）仅用于研究中。

可能产生之风险，副作用和不便之处:

- 使用电子容器（eCAPs）在易于使用及携带方面，可能会造成轻微的不便。
- 参与此研究的风险极微，您将接受新加坡全国眼科中心的标准青光眼护理。

预期效益

若您参与本研究，您可以合理地期望受益于研究中的一项或多项方法，以提高您按照剂量点滴青光眼药水的次数，进而有可能改善您的健康。此外，您的参与将有助于了解奖励对于坚持按照剂量点滴青光眼药水的有效性及成本效益。您也可能通过参与研究得到金钱及礼券津贴。

替代方案

若您选择不参与本研究，另一方法是进行被认为是您病情的标准护理。在新加坡全国眼科中心其为由眼科医生进行的例常检查、并开治疗青光眼药水的处方及若发现您不遵守用药时安排您进行青光眼辅导。

青光眼辅导有以下益处：

患者可了解治疗青光眼的有效方法，例如：了解青光眼、青光眼如何影响视野、风险因素、症状、青光眼管理、治疗、青光眼药物及药物的使用（例：自我点滴眼药水的技巧）。此外，辅导护士也将讨论药物的最佳使用时间、强调不遵守用药的风险及按照患者的生活方式与工作时间拟定出适合患者用药时间。

参与者权益

您在本研究中的参与全属自愿性质。您的问题将被清楚地为您解答，直到您满意为止。

若发现任何新资讯有可能会影响您继续参与研究的意愿时，您或您的法定代理人会通过研究总负责人或其代表被即时告知。

您不会因为签署这份知情同意书及参与本研究而放弃您的合法权益, 您仍可以在任何时候撤销您的同意，退出研究。

研究和医疗记录的保密性

本研究中所收集的任何讯息都将被视为机密。在相关的法律规范下，您的档案将不会对外公开。只有本研究的相关负责人及研究团队（杜克-新加坡国大医学研究生院及新加坡眼科研究中心）有权检视您所提供的机密资料。

虽然如此,监管机构、伦理审查委员会及卫生署亦将被授权在不公开您任何资料的前提下直接地审视您的原医疗记录，藉以审阅研究的程序以及资料。

若您或您的法定代表签署于此附上的知情同意书，亦表示您已授权我们（i）收集、获取、使用、储存及(ii)向服务提供商及相关第三方透露您的“个人资料”。

“个人资料” 是指能识别您的（i）有关您的资料，或（ii）机构所拥有或能得到资料或其他信息。其包括病情、使用的药物、检察及治疗史。

未来研究基于此个人资料保护政策，将需通过相关伦理审查委员会的审视。

参与本研究，亦表示您已阅读、理解并同意接受新加坡保健集团资料保护政策，完整版可在www.singhealth.com.sg/pdpa 查阅。打印件应要求而提供。

研究里所收集与输入病例表的数据都归杜克-新加坡国大医学研究生院所拥有，若作出任何有关本研究的发表，您的身份仍将保密。

参与研究的费用

若您参与本研究，以下将免费为您进行：提供及替换电子容器（eCAPs）

若您参与本研究，一下需您自费：所有医生看诊及研究期间所需的青光眼药物

我们将为您付出的时间，研究带给您的不便，与交通费用给于津贴， 如下：

- 若您完成研究，您将一共获得＄60
- 若您因任何原因而没有完成研究，您将获得如下的津贴：
  - 符合研究条件并完成研究基线问卷， 您可获得＄10
  - 在第 3 个月归还您的电子容器（eCAPs）， 您可获得$20
  - 在第 6 个月完成第 6 个月测试、归还电子容器（eCAPs） 及完成第 6 个月问卷，您可获得$30

研究相关的损伤及赔偿

本医院并没有做出任何规定以补偿研究参与者与研究有关的损伤，但由非疏忽原因造成的损伤的赔偿，将依据个别案件斟酌处理。

您不会因为签署这份知情同意书而放弃您的合法权益，或豁免各方在本研究中因疏忽负上的法律责任。

相关联络人

若您在参与本研究的过程中对于研究有任何疑问，或发生与研究有关的任何损伤，您可在办公时间（早上 8.30 至下午 6）联络研究总负责人 Marcel Bilger 电话： [redacted]，或 [redacted]电话： [redacted]

若您对参与研究或参与研究的相关权益有任何疑问，您可在办公时间（早上 8.30 至下午 5.30）联系审查及批准本研究的伦理审查委员会，电话 [redacted]。

本研究经由新加坡保健集团中央伦理审查委员会审查及批准。

若您对研究参与者的相关权益有任何疑问，您可在办公时间（早上 8.30 至下午 5.30）联系伦理审查委员会，电话 [redacted]。

若您有任何关于本研究的投诉，您可以联系研究总负责人或新加坡保健集团中央伦理审查委员会。

研究参与者知情同意书

研究详情

研究名称： 研究以奖励鼓励更好地按照剂量点滴青光眼药水

A Randomized Study on Economic Incentives for Glaucoma Medications Adherence (“SIGMA”)

研究总负责人:

Marcel Bilger

研究分支总负责人

Tina Wong 副教授

参与者个人资料

名字: ______________________ 身份证号码: ______________________

地址: ____

性别: 女 / 男 出生日期: ______________ 种族: 华/巫/印 其他__________

日/月/年 (请注明)

我, ______________________ (身份证/护照号码:______________)

(患者姓名)

同意依据参与者信息表所描述的条款参与以上的研究。

我已充分地讨论与了解本研究的程序及目的，也获得一份研究参与者信息表，有机会提出疑问,亦已得到满意的答案。

我了解我于本研究的参与是自愿性的，并可以在不需任何理由，在任何时间，在不影响我应有的医疗照顾的情况下自由地退出此研究。

参与本研究，我确认我已阅读、理解并同意接受新加坡保健集团资料保护政策。我也同意我的个人资料，使用于未来相关研究。

______________________________ ______________________

[参与者签名/ 指印(右/左)] (签名日期)

患者家长/ 监护人填写栏 （若有需要）

我 ______________________ 同意上述参与者参与此研究计划。我清楚地明白

(家长 / 监护人)

此研究的本质，风险以及好处。

______________________________ ______________________

[家长/监护人签名/ 指印(右/ 左)] (签名日期)

翻译者资料（若有需要）

______________________________已通过 ________________________________

翻译者姓名　　　　　　　　　　　　语言

向参与者或其法定代理人解释此研究的性质。

见证人填写栏（若有需要）

在患者或患者的法定代理人无法阅读的情况下，必须有一位公正的见证人在场见证整个签署和讨论知情同意书的过程。在书面同意书及任何书面资料已向患者或其法定代理人口头转述与解释之后，并且患者或其法定代理人已经口头同意参与本研究，及患者或其法定代理人有能力并已经亲自签署同意书并附上日期的情况下，见证人也必须亲自签署此栏并附上日期。

见证人: ____ _________________________________

（见证人名字）　　　　　　　　　　（见证人身份）

____ ________________________________

［见证人签名］　　　　　　　　　　（签名日期）

研究负责人声明

我在此签名,并证明以我所知，这份同意书的签署人/签署人的法定代理人已经通过详尽的解释，清楚地了解他/她 /他/她的受监护人参加本研究的性质，风险和效益。

_________________________ ___________________________ _________________________

（研究负责人姓名）　　　　（研究负责人签名）　　　　（签名日期）
